# IL-15 enhances the anti-tumor activity of trastuzumab against breast cancer cells but causes fatal side effects in humanized tumor mice (HTM)

**DOI:** 10.18632/oncotarget.13159

**Published:** 2016-07-11

**Authors:** Anja K Wege, Florian Weber, Alexander Kroemer, Olaf Ortmann, Falk Nimmerjahn, Gero Brockhoff

**Affiliations:** ^1^ Department of Gynecology and Obstetrics, University Medical Center Regensburg, 93053 Regensburg, Germany; ^2^ Institute of Pathology, University Hospital Regensburg, 93053 Regensburg, Germany; ^3^ MedStar Georgetown Transplant Institute, Georgetown University Hospital, Washington, DC, USA; ^4^ Institute of Genetics, Department of Biology, University of Erlangen-Nuremberg, 91058, Erlangen, Germany

**Keywords:** humanized tumor mice (HTM), trastuzumab, IL-15, immunomodulation, breast cancer

## Abstract

Cancer immunotherapy has been shown to enhance established treatment regimens. We evaluated the potential reinforcing effect of IL-15 in trastuzumab treated humanized tumor mice (HTM) which were generated by concurrent transplantation of neonatal NOD-scid IL2Rγnull mice with human hematopoietic stem cells (HSC) and HER2 positive breast cancer cells (metastasizing SK-BR-3, solid tumor forming BT474).

We found that trastuzumab treatment efficacy mainly depends on the immediate anti-tumorigenic cellular effect which is significantly enhanced by tumor interacting immune cells upon cotransplantion of HSC. However, trastuzumab treatment caused elevated CD44 expression on tumor cells that metastasized into the lung and liver but did not hinder tumor cell dissemination into the bone marrow. Moreover, in a number of SK-BR-3-transplanted animals disseminated CD44^high^/CD24^low^ tumor cells lost trastuzumab sensitivity. Concerning the FcγRIIIa polymorphism, trastuzumab treatment efficiency in HTM was higher in mice with NK-cells harboring the high affinity FcγRIIIa compared to those with low affinity FcγRIIIa. In contrast, IL-15 caused the strongest NK-cell activation in heterozygous low affinity FcγRIIIa animals. Although IL-15 enhanced the trastuzumab mediated tumor defense, an unspecific immune stimulation resulted in preterm animal death due to systemic inflammation. Overall, treatment studies based on “patient-like” HTM revealed critical and adverse immune-related mechanisms which must be managed prior to clinical testing.

## INTRODUCTION

Antigen-specific antibodies are the primary tool for individualized treatment of cancer patients. Specific tumor targeting has a high curative potential and is typically associated with less systemic side effects compared to cytotoxic treatment regimens. The treatment of early and advanced HER2-positive breast cancer (BC) patients with trastuzumab (Herceptin®), a humanized monoclonal anti-HER2 antibody, results in both prolonged disease-free and overall survival (DFS, OS). However, >50% of HER2-positive patients do not benefit due to of *de-novo* or acquired resistance [1]. On the one hand, however, therapy failure has been attributed to cellular effects (e.g., inefficient trastuzumab binding or activation of alternate signaling pathways). On the other hand there is apparently an insufficient activation of immune effector cells, e.g., NK-cells and macrophages, which are thought to exert antibody-dependent cellular cytotoxicity (ADCC) [1].

The potential impact of an ADCC-related immune defense triggered by trastuzumab has been discussed controversially for many years. For example, Clynes et al. reported increased tumor growth in FcgRIII knock down mice [2]. Barock and colleagues demonstrated loss of function in trastuzumab-Fab compared to the native Fc containing immunoglobuline [3]. Moreover, a delayed progression of trastuzumab-treated BC disease has been linked to increased NK-cell tumor infiltration and enhanced ADCC [4-7]. In contrast to the aforementioned findings the therapeutic expansion and activation of NK-cells in patients by IL-2 administration did not enhance immunological tumor defense or improve outcome [8]. Other clinical studies revealed a beneficial effect of ADCC only in a monotherapeutic treatment setting but not in combination with chemotherapy [9]. However, Petricevic et al. reported that efficacy of trastuzumab-specific ADCC was not affected by treatment duration, disease progression or concomitant chemotherapy [10]. Overall, the impact of trastuzumab-triggered ADCC on therapy success in BC patients remains unclear.

Nevertheless, the presence of tumor infiltrating lymphocytes (TILs), which include T- NK- and other cells, has been associated with a favorable outcome in HER2-positive (and triple negative) BC patients [11-12], although, tumor cells develop a variety of mechanisms to avoid immune defense. A number of escape mechanisms are known to affect NK-cell activity, e.g., the secretion of immunosuppressive cytokines (e.g. TGFb) [13], the induction of regulatory T- [14] or myeloid derived suppressor cells (MDSC) [15], the expression of programmed death ligand-1 (PDL-1) [16] or first apoptosis signal (FAS) ligand [17], the induction of Indolamin-2,3-Dioxygenase (IDO) [18], and the secretion of soluble MHC class I chain-related (MIC) molecules MICA/B [19].

Thus, a potential approach to overcome the immunosuppressive activity of tumor cells is cytokine-mediated immune (especially T- and NK-) cell activation. IL-15 is known to stimulate NK-cells both *in-vitro* [20] and *in-vivo* [21-23]. The therapeutic potency of IL-15 in advanced melanoma and renal cell cancer patients [24] has been investigated in previous clinical trials. However, side effects which were not recognized in previous clinical studies performed in primates (rhesus macaque) [25], forced dosage reduction. Subsequently, investigations based on recombinant human IL-15 (rhIL-15) and IL-15 receptor complex (IL15Rα) have been initiated to evaluate the maximum-tolerated dose and an efficient application route. The results of these studies, however, are still pending.

In this context, we assessed the therapeutic efficiency of IL-15 to boost the therapeutic activity of trastuzumab in HTM, which were generated by the cotransplantation of HSCs and HER2-positive BT474 and SK-BR-3 BC cells into neonatal immunodeficient NSG mice which resulted in two different HTM models: The transplantation of only moderately trastuzumab sensitive SK-BR-3 cells results in an ascitis with greater incidence of metastases in different organs including the brain. In contrast highly trastuzumab sensitive BT474 cells form a solid tumor growth upon transplantation with fewer metastases and no dissemination into the brain. Based on these different HTM models, we investigated the immune response, the importance of FcgRIIIa polymorphism, and the adaptation processes of the tumor cells during trastuzumab and IL-15 treatment.

## RESULTS

### Trastuzumab treatment prolongs DFS and OS in BT474 but not in SK-BR-3-based HTM

**Figure 1:**
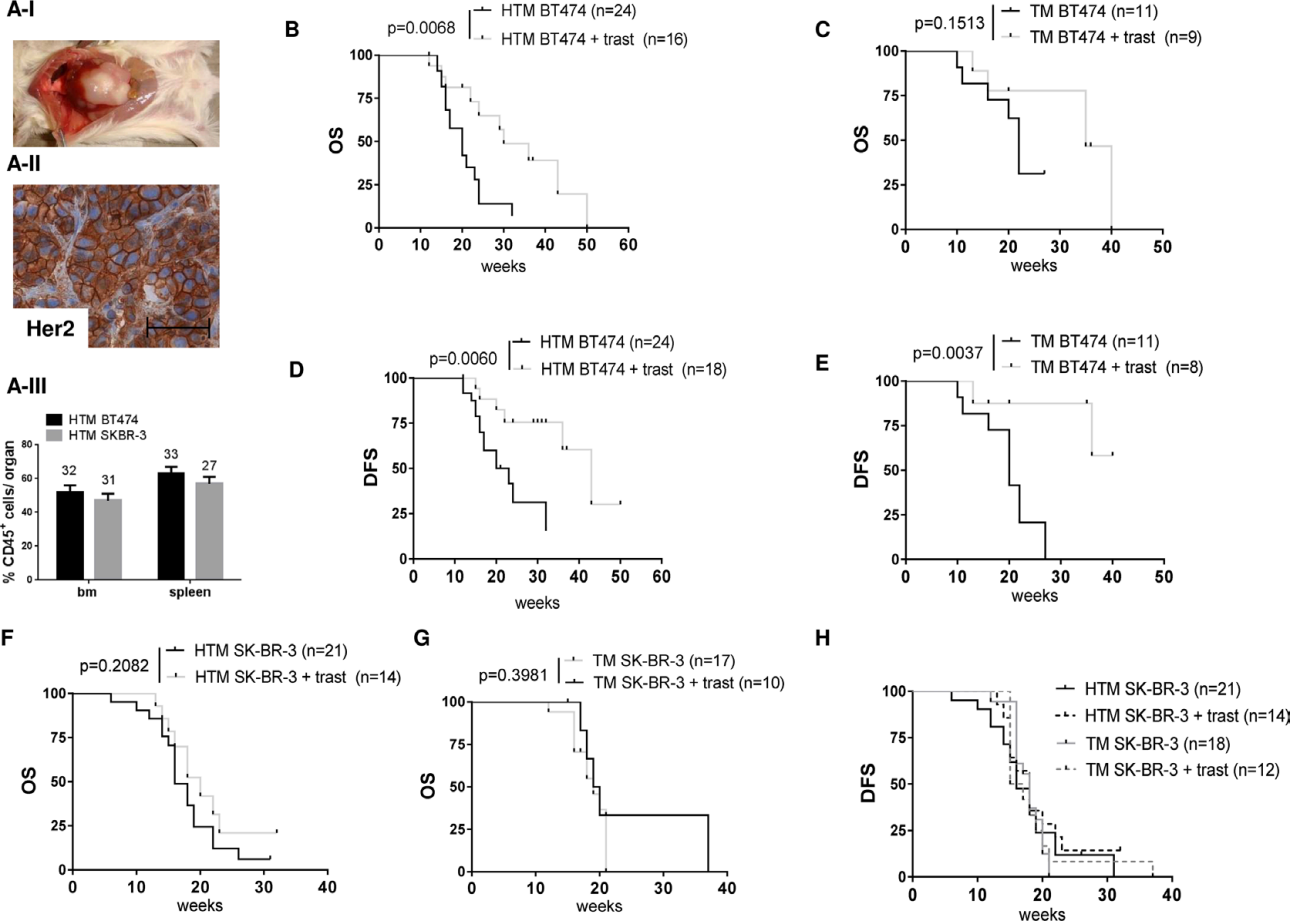
The overall and tumor-free survival in BT474- and SK-BR-3-transplanted tumor mice is differently influenced by anti HER2 (trastuzumab) treatment **(A-1)** BT474-transplanted HTM develop liver-associated tumors with HER2 over expression **(A-II)** Scale bar represents 100 μm. Flow cytometric analyses reveal an equivalent percentage of human engraftment (% CD45^+^ cells) in the spleen and bone marrow (bm) of SK-BR-3- and BT474-transplanted HTM **(A-III)** The number of animals included in each analysis is mentioned above each bar. Illustration of overall survival (OS) and tumor-free survival (TFS) of BT474-transplanted HTM **(B, D)** and TM **(C, E)** and SK-BR-3-transplanted HTM **(F, H)** and TM **(G, H)** respectively. Significance was calculated using the Log-rank (Mantel-Cox) test, and the number of analyzed animals is indicated in brackets.

Upon the simultaneous transplantation of human CD34^+^ hematopoietic stem cells (HSC) and human BC cell lines (BT474 and SK-BR-3), the NSG mice developed liver-associated tumor growth (BT474; Figure 1A-I) or tumor cell effusion in the peritoneal cavity (SK-BR-3). IHC verified the preservation of HER2 over-expression in the originally transplanted tumor cells (Figure 1A-II). The amount of human reconstitution (% human immune cells) in both models was similar (Figure 1A-III) and therefore independent of the co-transplanted tumor cell line (BT474 spleen: 63 +/-4 SEM; BT474 bone marrow (bm): 52 +/- 4; SK-BR-3 spleen: 57+/- 4, SK-BR-3 bm: 47+/-4). Further analyses of cell subsets revealed an increased proportion of B cells in the spleen of SK-BR-3 (Supplementary Figure S1A; average age 16 weeks post transplant) compared to BT474 transplanted mice (Supplementary Figure S1B; average age 23 weeks post transplant) which is due to the different time point of analyses. The courses of T-cell (increase over time) and B-cell (decrease) fraction sizes over time were monitored in all HTM (Supplementary Figure S1C). The overall percentage of human immune cells (CD45) and the myeloid cell (CD33) fraction remained considerable stable within 10 - 43 weeks of the animals’ life time (Supplementary Figure S1C). Tumors were detectable in BT474 and SK-BR-3 transplanted animals sacrificed 10 weeks post transplant.

To investigate the impact of trastuzumab on solid tumor growth, BT474 transplanted NSG mice were treated once a week and the OS of the animals was evaluated (Figure 1B). The OS of HTM was significantly prolonged by the trastuzumab treatment (Figure 1B; p=0.0068), whereas the trastuzumab-treated TM without a human immune system did not reach a statistically significant survival benefit (Figure 1C; p=0.1513). The histological examination also revealed reduced lung metastatic diseases in HTM but not in TM (Table 1A), reflecting the metastasis-impairing effect of the human immune system. However, the DFS was prolonged by trastuzumab treatment both in the presence (HTM; Figure 1D) and absence (TM; Figure 1E) of the immune system. Since SK-BR-3 cells were isolated from a patient's effusion, these cells recapitulate their original growth behavior in the form of a peritoneal -located ascites in NSG mice. The development of ascites accompanies cell metastasis into different organs including the brain and bm. Remarkably, trastuzumab treatment of SK-BR-3-transplanted mice does not improve either the OS (Figure 1F & 1G) or DFS (Figure 1H) of these animals. Apparently, there is no efficient immune cell activity to compensate for trastuzumab inefficacy, although reduced brain metastasis could be observed in humanized SK-BR-3-based HTM (p=0.0179) but not in TM (p=1; Table 1B).

**Table 1:** Immunohistological assessment of metastases in trastuzumab-treated and non-treated (control) HTM and TM

A. BT474						
	HTM control	HTM + trast	p-value (control versus trast)	TM control	TM + trast	p-value (control versus trast)
lung	10/19	1/12	**0.0201**	4/8	3/11	0.3765
liver	6/13	1/3	1	4/10	2/8	0.6380
spleen	2/17	0/11	0.5053	0/6	0/8	1
brain	0/10	0/9	1	1/4	0/8	0.3333
**B. SK-BR-3**						
	**HTM control**	**HTM + trast**	**p-value (control versus trast)**	**TM control**	**TM + trast**	**p-value (control versus trast)**
Lung	13/14	8/8	1	11/11	4/4	1
Liver	7/12	7/8	0.3246	9/11	3/3	1
spleen	6/11	3/5	1	9/11	4/4	1
brain	5/5	0/3	**0.0179**	7/8	1/1	1

BT474 (A) and SK-BR-3 (B) transplanted HTM and TM were immunohistologically stained using anti-HER2 antibodies in the lung, liver, spleen and brain. The number of animals with detectable HER2^+^ metastasis of the total number of animals (n/n) is indicated. Statistical differences were calculated using the two-sided Fisher's exact test and significant differences are marked in bold.

### Adjuvant IL-15 administration in combination with trastuzumab diminishes tumor burden but also reduces DFS and OS in BT474 and SK-BR-3 HTM

**Figure 2:**
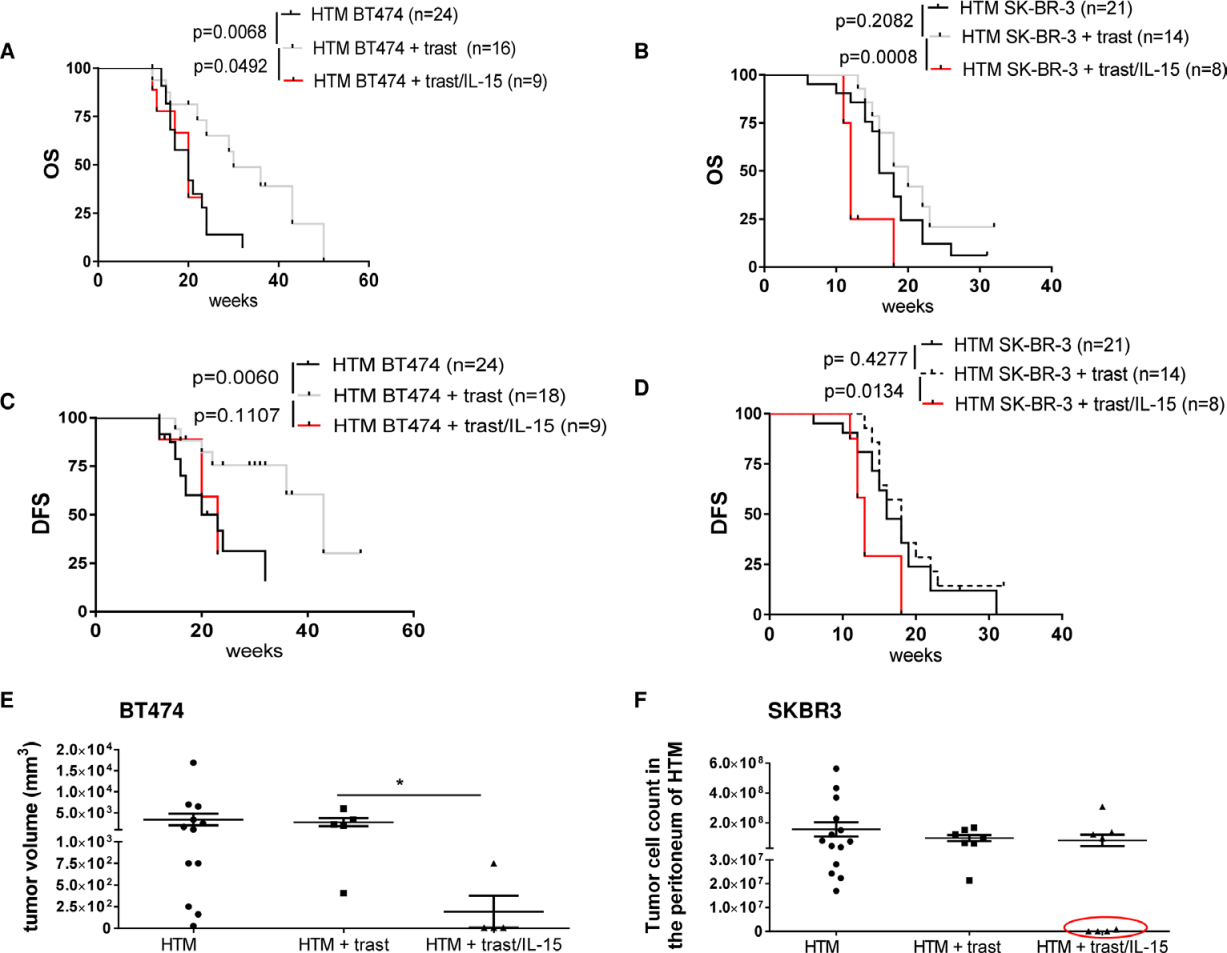
IL-15 immunostimulation influences the outcome of trastuzumab treatment in HTM Graphs represent the OS and TFS in BT474 **(A, C)** and SK-BR-3 **(B, D)** transplanted HTM treated with trastuzumab +/- IL-15. Significance was calculated with the Log-rank (Mantel-Cox) test. **(E)** Tumor volume of treated and untreated BT474 HTM. Significance was calculated with Tukey's multiple comparisons test (*=p<0.05). **(F)** Tumor cell burden was counted in SK-BR-3-transplanted HTM in the peritoneum (ascitis) of the animals. HTM with low or no tumor cell count are marked with a red circle.

HTM received the potent IL-15/IL-15Rα complex every two weeks. Unexpectedly, the systemic administration of IL-15 in combination with trastuzumab caused a significant reduction in OS in both the BT474- (Figure 2A) and SK-BR-3- (Figure 2B) based HTM model. The DFS was not reduced in BT474 HTM (Figure 2C) but was reduced in SK-BR-3 HTM (Figure 2D). In BT474 HTM, the tumor volume in tumor-bearing animals shrank from 3416 mm^3^ (+/- 1401, n=12) in control mice to 2800 mm^3^ (+/- 932; n=5) in trastuzumab-treated animals down to 192 mm^3^ (+/- 186; n=4) in IL-15/trastuzumab-treated mice (Figure 2E). Similarly, 50% (4/8) of the treated SK-BR-3 HTM had no, or only a very low level of tumor burden left in the peritoneum upon IL-15/trastuzumab treatment (Figure 2F), whereas trastuzumab therapy alone was not sufficient to reduce the amount of tumor cells in any of the treated mice. In addition, the tumor burden in the lung of IL-15/trastuzumab-treated mice tended to be lower compared to control or trastuzumab-treated HTM (Supplementary Figure S2A & S2B).

**Figure 3:**
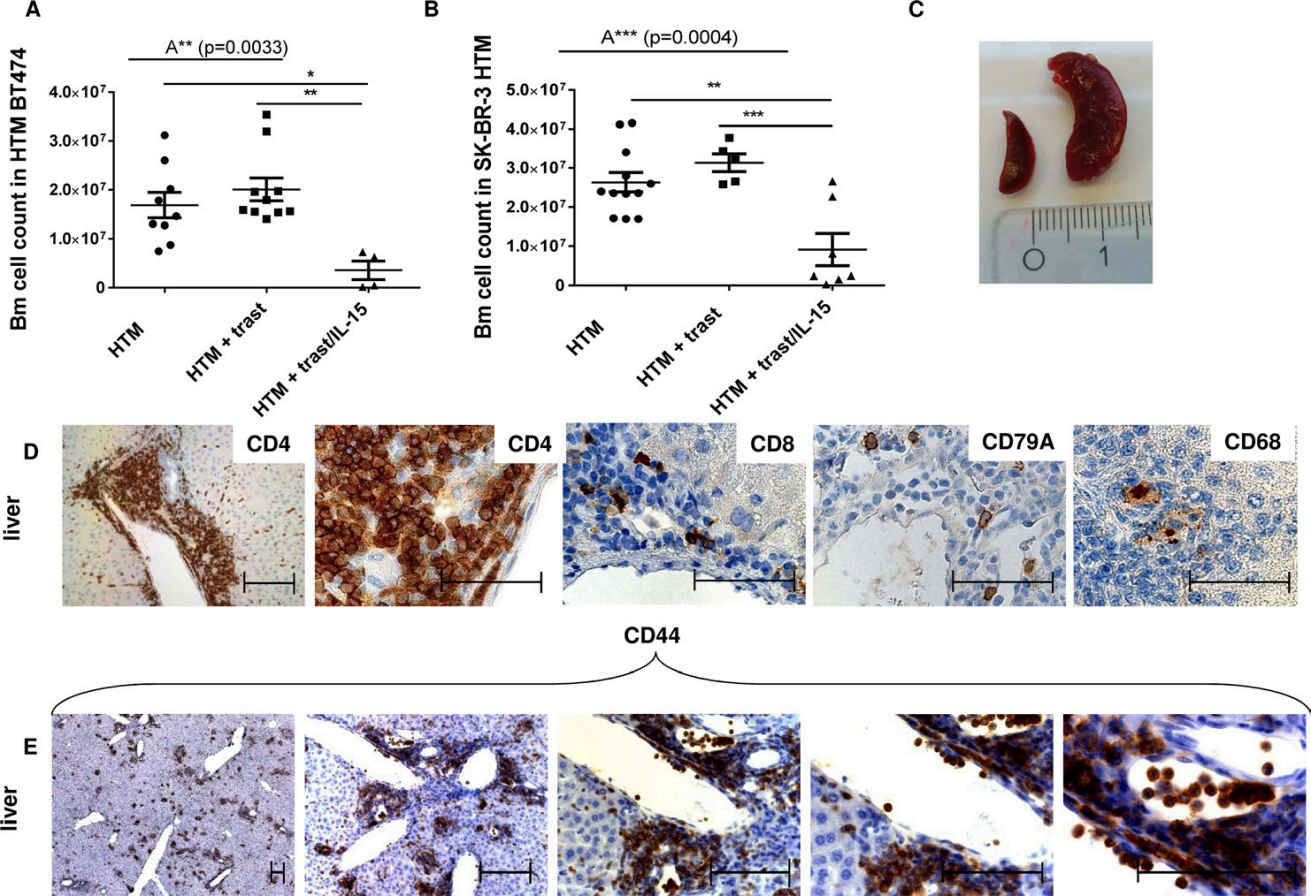
Side effects induced by IL-15 treatment in HTM Bone marrow (bm) cell counts from femur of BT474 **(A)** and SK-BR-3 **(B)** transplanted HTM are illustrated. Significance was calculated using the One-way Anova (A**, A***) and Tukey's multiple comparison test (*=p<0.05; **=p<0.01; ***=p<0.001). **(C)** Image of a splenomegalie of an IL-15-treated HTM (right spleen) compared to an untreated HTM (left spleen). **(D+E)** Immunohistological staining of lymphocyte population in the liver of an IL-15-treated HTM (CD4 = T helper cells; CD8 = cytotoxic T-cells, CD79A = B-cells; CD68 = macrophages; CD44 = activated T-cells). Bars represent 100 μm.

The considerably reduced OS in both xenograft types (primary solid tumor growth and primary effusion) strictly correlates with (i) an immune cell depletion observed in the bm (Figure 3A & 3B), (ii) with a splenomegaly (Figure 3C), and (iii) with immune cell invasion into various organs (Figure 3D; liver exemplarily shown). Moreover, the liver (Figure 3E) and lung infiltrating leucocytes highly expressed CD44, thus indicating their activation and capacity for recirculation and homing. CD44-positive leucocytes were also found in the spleen of non-treated control HTM but were not detectable in the tumor or liver of these animals (Supplementary Figure S3). In trastuzumab-treated HTM, CD44 expression was triggered in leucocytes located in the spleen but it was only rarely visible in the tumor tissue or in other organs (e.g. liver; Supplementary Figure S3). CD44 expression in IL-15/trastuzumab treated HTM was increased in spleen, tumor, and liver (Supplementary Figure S3).

**Figure 4:**
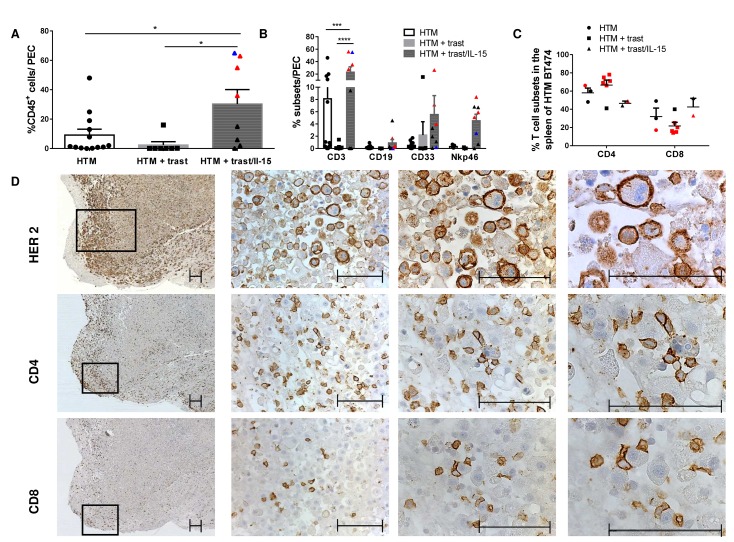
Tumor infiltrating lymphocytes are increased after IL-15 stimulation Graphs represents the percentage of human leucocytes (%CD45; A) and the immune subpopulations (T-cells (CD3), B-cells (CD19), myeloid cells (CD33), and NK-cells (Nkp46)) in the peritoneal exudate cells (PEC; A, B). Red symbols represent HTM without and blue symbols represent HTM with few remaining tumor cells in the peritoneum. Significance (*=p<0.05; ***=p<0.001; ****=p<0.0001) was calculated using Tukey's multiple comparison test **(A)** and Bonferoni's multiple comparison test **(B+C)** and values were included in the graphs when p was below 0.5. **(C)** Graphs summarize CD4 and CD8 distribution in the spleen of BT474 HTM. Red symbols represent HTM without detectable tumors at the end of the experiments. **(D)** Immunohistological staining of HER2, CD4 and CD8 in a tumor sample of SK-BR-3-transplanted and IL-15-treated HTM. Bars represent 100 μm.

Moreover, IL-15 treatment significantly stimulated a human immune cell infiltration into the tumor cell loaded peritoneum of SK-BR-3 HTM (Figure 4A). Advanced flow cytometric analysis revealed that the main population of invasive immune cells belonged to the CD3^+^ T-cell subset (Figure 4B). Particularly tumor-free HTM (red symbols) showed an increased T-cell infiltration but also enhanced NK- and myeloid cell influx (Figure 4B). The main population of TILs in solid tumors consisted of CD4 T-cells in both mouse models (Figure 4D exemplarily shown for SK-BR-3 HTM). Notably, HTM without detectable solid tumor formation (BT474, red symbols) at the end of the experiments showed a tendency towards a higher CD4 T-cell fraction in the spleen compared to tumor-bearing mice (Figure 4C).

### CD44 expression in tumor cells is caused by trastuzumab treatment *in-vivo* and is increased in trastuzumab-resistant disseminated tumor cells isolated from the bm

The CD44, CD24, HER2, and epithelial cell adhesion molecule (EpCAM) expression on BT474 and SK-BR-3 tumor cells was assessed by flow cytometry as a function of tumor treatment (Supplementary Figure S4A). We found a significantly increased CD44 expression on metastasized tumor cells in the lung and the liver of HTM and TM with trastuzumab treatment (Supplementary Figure S4B). However, the EpCAM (data not shown) and CD24 expression (Supplementary Figure S4C) was not altered. Tumor cells isolated from ascites (i.e., peritoneal tumor cells, PTC) did not show a significant change in any of the tested markers (Supplementary Figure S4B & S4C).

**Figure 5:**
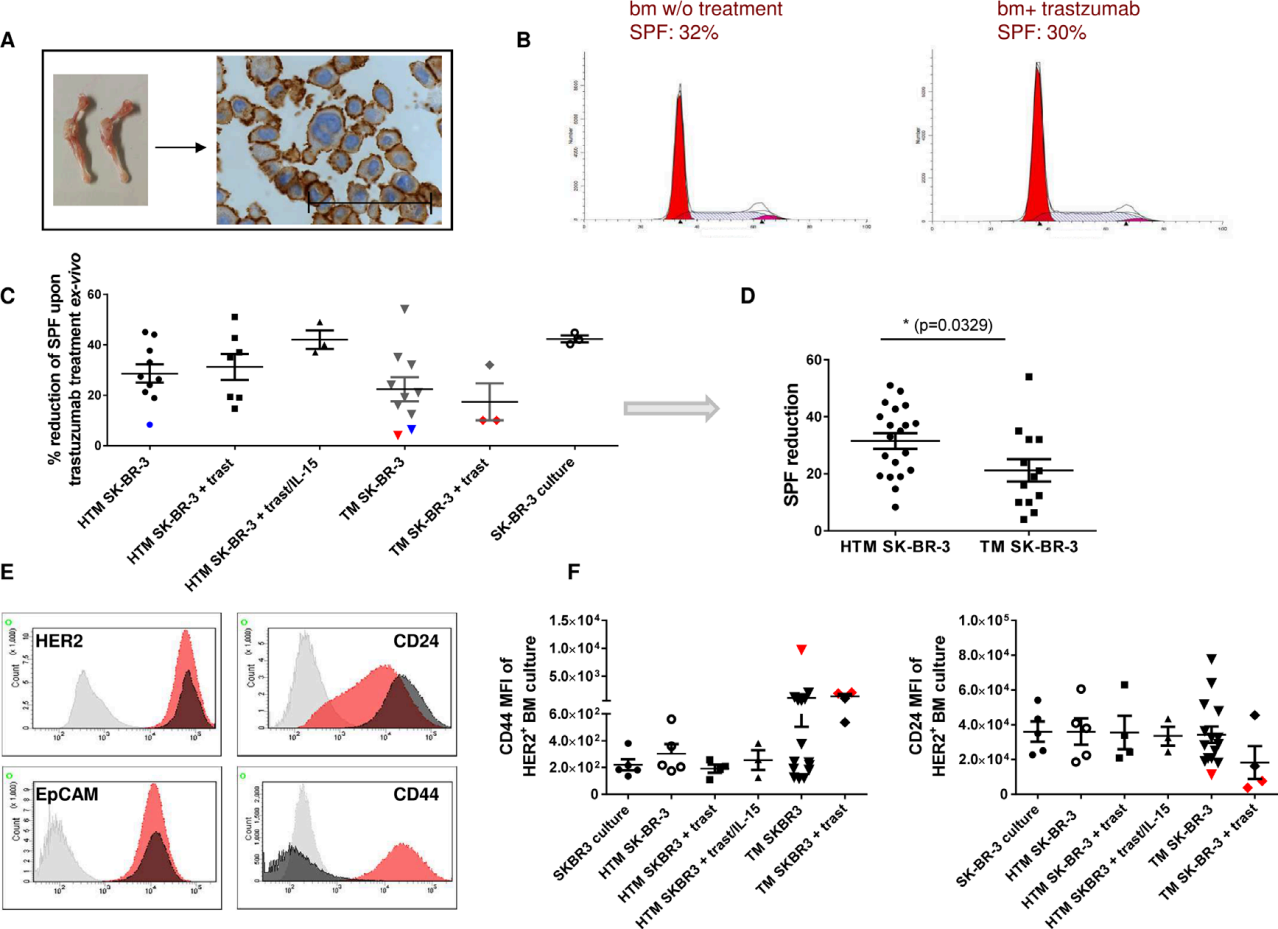
Trastuzumab resistance in ex vivo expanded DTC from the bone marrow is associated with increased CD44 expression in SK-BR-3-transplanted NSG **(A)** SK-BR-3-expanded DTC cell cultures extracted from femur and tibia (stained with anti-HER2; scale bar represents 100 μm) were incubated in the presence or absence of trastuzumab for 48 hours. The S-phase fraction (SPF, grey) of treated and untreated cultures did not significantly differ and is presented in Figure 5 (B) (30% vs. 32%). A collection of all data is illustrated in Figure 5 (C) and summarized for HTM and TM (independent of treatment) in **(D, E)** Exemplary histograms of HER2, CD24, EpCAM, and CD44 expression of the isotype (grey), SK-BR-3 cell culture (black) and one trastuzumab insensitive bone marrow (bm) culture (red) are shown. **(F)** Mean fluorescence intensity (MFI) of CD44 and CD24 expression in HER2^+^ tumor cells from ex-vivo expanded bm cultures are illustrated in the graphs. Symbols related to trastuzumab insensitive animals who were also analyzed for CD44 & CD24 expression, are displayed in red (C&E). Resistant cells without corresponding CD44/CD24 data are marked in blue. No significant differences were detectable in C & F using Tukey's multiple comparison test. Statistical differences were calculated using the two-tailed unpaired t test in D (p = 0.0329).

Cancer metastasis is the main cause of cancer-related death and disseminated tumor cells (DTCs), preferably homing in the bm niche, are considered to be the major source of metastasis. To characterize DTCs in the bm of HTM, single cells from the bm aspirates were expanded *ex-vivo* (Figure 5A; HTM: 13.3 weeks +/- 1.5, n=17; TM: 10.1 +/- 1.2, n=18) and subsequently analyzed by flow cytometry. The propagation of bm-derived DTCs was more successful in samples derived from SK-BR-3-transplanted mice than from BT474-transplanted mice (Table 2). The cell propagation was not affected by the presence of immune cells (HTM versus TM; BT474: p = 1; SK-BR-3: p = 0.16; two-sided Fisher's exact test) or by exposure to trastuzumab (Table 2; BT474: p = 1 (HTM & TM); SK-BR-3: p = 0.7 (HTM) & 0.6 (TM)). Combined trastuzumab/IL-15 treatment tended to reduce the number of successful *ex-vivo* DTC cultures. In addition, we assessed the fraction of proliferating SK-BR-3 cells (S-phase fraction, SPF) derived from HTM and TM bm aspirates with or without trastuzumab treatment. A flow cytometric DNA profile of resistant cells is presented in Figure 5B. A total of 5 trastuzumab insensitive bm cultures were obtained from SK-BR-3 cell transplanted TM (n = 4) and HTM (n = 1) in both treated and untreated animals (Figure 5C). Cumulatively, the reduced sensitivity is more pronounced in samples derived from TM than from HTM (Figure 5D). This observation is independent of treatment regimen.

**Table 2:** Rate of ex-vivo propagated DTCs derived from the bm of BT474 and SK-BR-3-transplanted HTM and TM

treatment/mice	No treatment (*control*)	Trastuzumab *(+trast*)	p-value (*control versus trast*)	Trastuzumab +IL-15 (*trast/IL-15*)	p-value *(trast versus trast/IL-15)*
HTM BT474	4/19 (21%)	3/14 (21%)	1	0/4 (0%)	1
TM BT474	2/9 (22%)	1/7 (14%)	1	---	--
HTM SK-BR-3	12/20 (60%)	10/14 (71%)	0.7170	3/8 (38%)	0.1870
TM SK-BR-3	16/19 (84%)	9/12 (75%)	0.6526	---	--

Single cells isolated from both femurs of BT474 and SK-BR-3-transplanted HTM and TM were cultured to expand DTC. The number of animals with successfully expanded DTC cultures of all tested samples (n/n) is indicated. Statistical differences were calculated using the two-sided Fisher's exact test.

Different bone marrow cultures were phenotyped for HER2, CD24, CD44, and EpCAM (presented in Figure 5E). Notably, all tested resistant cell samples showed enhanced CD44 and reduced CD24 expression (red symbols, Figure 5F). 6/16 bm aspirates derived from TM remained sensitive to trastuzumab treatment in spite of an elevated CD44 expression.

### FcgRIIIa polymorphism affects the *in-vivo* treatment efficiency of trastuzumab both in the presence and absence of IL-15 in HTM

Low and high binding affinity of IgGs to FcgRIIIa is due to a receptor polymorphism, which is supposed to be involved in enhanced or poor NK-cell activation by therapeutic antibodies. Therefore, we analyzed the most common genotypes (homozygous low affinity (F/F) and the heterozygous F/V (low/high affinity) in HTM.

**Figure 6:**
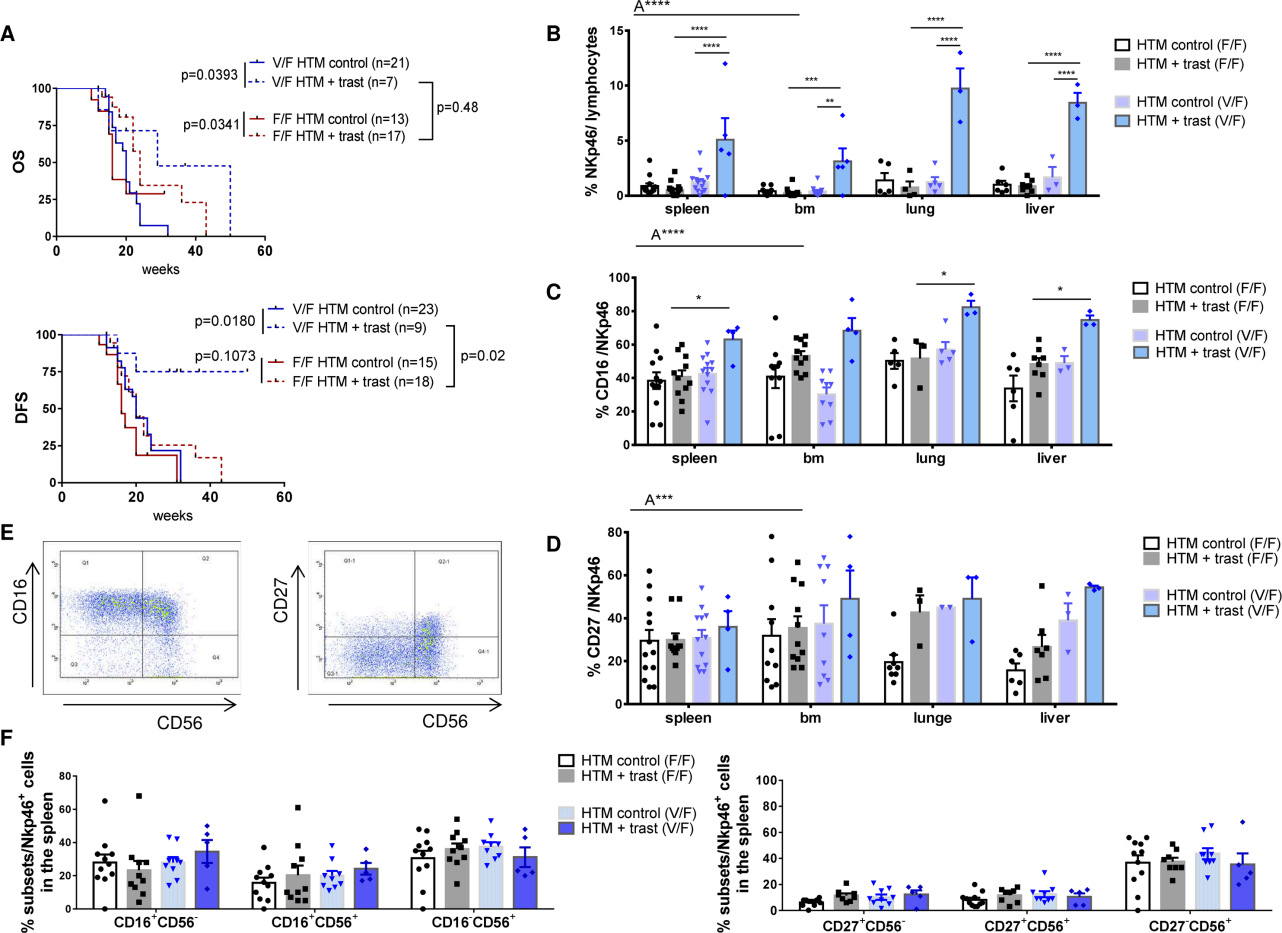
Influence of the FcgRIIIA genotype on NK-cell population and the overall outcome in trastuzumab-treated HTM **(A)** Illustration of OS and DFS of BT474 and SK-BR-3-transplanted HTM dependent on the hetero (V/F) or homozygous (F/F) low affinity FcgRIIIA genotype. Numbers of analyzed animals are indicated in brackets. Significance was calculated using the Log-rank (Mantel-Cox) test. Flow cytometric analyses of the overall percentage **(B)** and the percentage of CD16 expression **(C)** of NK-cells (Nkp46^+^) in the spleen, bone marrow (bm), lung and liver of treated and untreated HTM. Exemplary density plots of Nkp46 sub population expression CD16, CD56, and CD27 are shown in **(D)** and are summarized for all animals in **(E & F)** Differences were calculated using one-way (F) and two-way Anova (A**** = p <0.0001, A*** = p <0.001) and Tukey's multiple comparison test (* *= p <0.01; *** = p <0.001; **** = p <0.0001; B, C, D) and values were included in the graphs when p was below 0.5. Bars represent the mean +/- SEM and each symbol represents one single animal.

OS of both BT474- and SK-BR-3-based HTM was significantly prolonged by trastuzumab treatment independent of the FcgRIIIa genotype, whereas the DFS was only extended in mice transplanted with heterozygous (V/F) high affinity CD34^+^ HSC (Figure 6A). This pronounced responsiveness of the V/F bearing group is also reflected by the significantly increased NK-cell number (in between V/F control and trastuzumab; between trastuzumab V/F and trastuzumab V/V; Figure 6B). In addition, CD16 expression was significantly increased in the trastuzumab-treated high affinity (V/F) versus low affinity (F/F) FcgRIIIa genotype (Figure 6C). However, the expression of other NK-cell-related differentiation markers (CD27, CD56, Figure 6D/data not shown) and the distribution of NK-cell subpopulations (effector/regulatory subsets, Figure 6E & 6F) do not depend on the FcgRIIIa genotype. Nevertheless, the CD56^+^CD27^−^ phenotype represents the majority of NK-cells in the spleen (Figure 6F).

**Figure 7:**
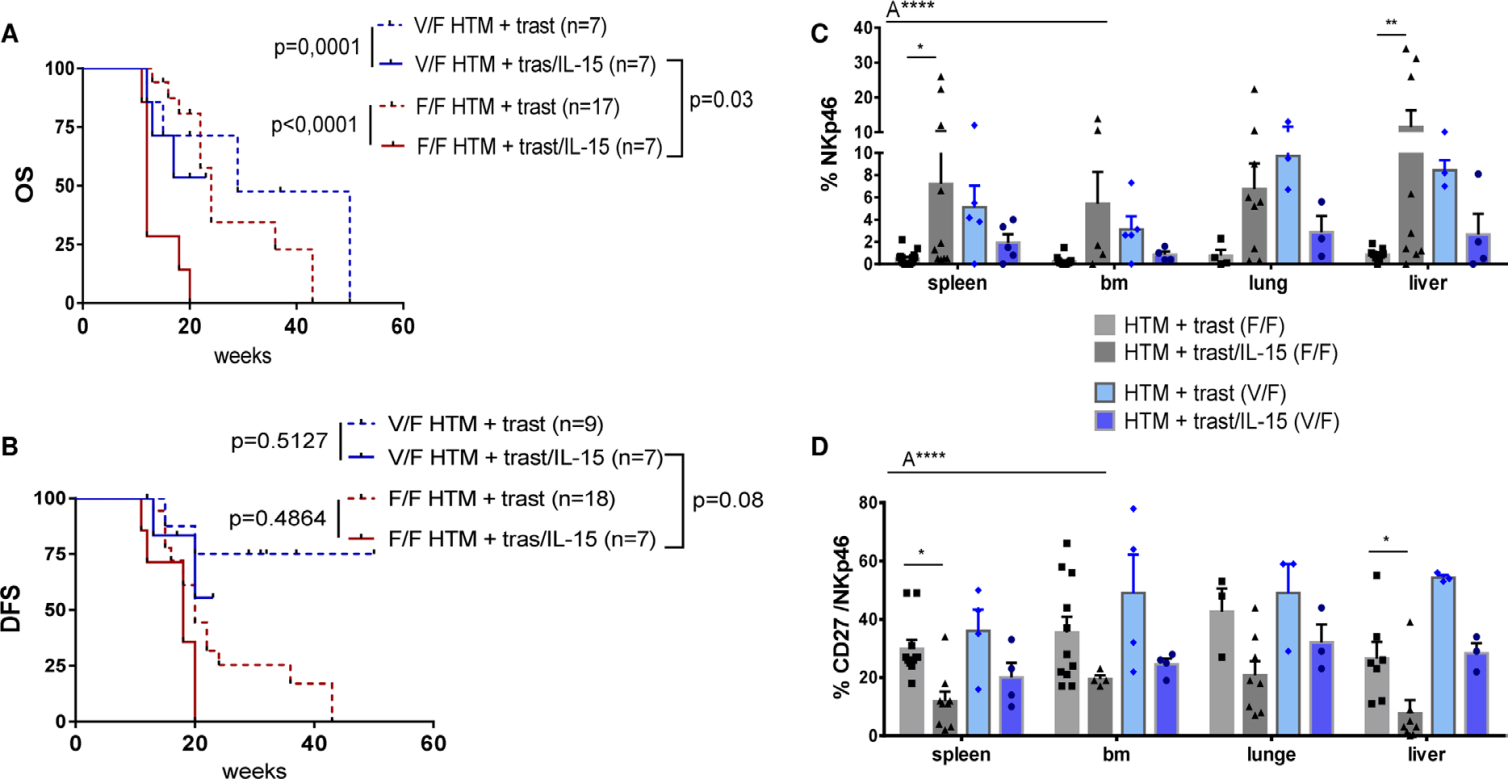
Influence of the FcgRIIIA genotype on NK-cell population and the overall outcome in IL-15-treated HTM Illustration of OS **(A)** and DFS **(B)** of BT474 and SK-BR-3 transplanted HTM dependent on the hetero (V/F) or homozygous (F/F) low affinity FcgRIIIA genotype. Numbers of analyzed animals are indicated in brackets. Significance was calculated using the Log-rank (Mantel-Cox) test. Flow cytometric analyses of the overall percentage **(C)** and the percentage of CD27 expression **(D)** of NK-cells (Nkp46^+^) in the spleen, bone marrow (bm), lung and liver of treated and untreated HTM. Differences were calculated using two-way Anova (A****=p<0.0001) and Tukey's multiple comparison test (*=p<0.05; **=p<0.01). Bars represent the mean +/- SEM and each symbol represents one single animal.

IL-15 stimulation reduced the OS in the presence of both the low and high affinity FcgRIIIa variant. This effect is even more pronounced when low affinity FcgRIIIa receptors (F/F) are expressed (Figure 7A). The DFS was not significantly affected by the IL-15 treatment or by the FcgRIIIa genotype (Figure 7B). The unfavorable effect of IL-15 treatment on OS in the heterozygous low affinity group (Figure 7A) was associated with an increased NK-cell number in the spleen and the liver of HTM (Figure 7C). Additionally, the CD27 expression in NK-cells was significantly reduced (Figure 7D). An association between a CD27 loss and an increased cytotoxic T-effector and NK-cell function has been previously reported [26-27]. The expression of CD16 and CD56 on NK-cells and the distribution of NK-cell subpopulations (effector/regulatory subsets) was independent from the FcgRIIIa genotype (data not shown). Notably, tumor eradication in HTM was observed in the presence of both the low and high FcgRIIIa genotype (SK-BR-3: n = 3 F/F, n = 1 V/F; BT474: n = 5 V/F, n = 1 F/F).

## DISCUSSION

Although trastuzumab significantly improves the overall outcome of BC patients, a significant fraction of patients suffer from *de-novo* or acquired resistance. Immune evasion of cancer cells is considered to be a major mechanism that accounts for therapy failure, even in antibody-treated patients. Here, we investigated the treatment efficiency of trastuzumab alone and in combination with IL-15 in HER2-positive BC-based HTM. Upon cotransplantation of HSCs and BC cells, the mice concurrently develop a human immune system and human tumor growth [23]. Thus, the HTM model facilitates the analysis of antibody-based and immunomodulatory tumor treatments under unique “human-like conditions.” We utilized two HER2 overexpressing BC cell lines and generated a BT474- and a SK-BR-3- based TM (w/o a human immune system) and HTM (humanized with hematopoietic stem cells), in which solid tumor growth and tumor cell effusion developed, respectively. Trastuzumab treatment efficiency was assessed in the presence and absence of IL-15, and as a function of FcgRIIIa polymorphism.

Trastuzumab-treated BT474-based HTM showed an increased OS and DFS compared to non-treated mice. In contrast, SK-BR-3-bearing mice were mostly trastuzumab insensitive which is in line with the response characteristic of SK-BR-3 cells *in-vitro* [28]. Apparently, the immune system enhanced the trastuzumab treatment efficiency in BT474 HTM. The rate of metastasis was reduced in the brain (SK-BR-3) and in the lung (BT474) in HTM but not in TM. Obviously, the success of trastuzumab treatment *in-vivo* depends on both the immediate sensitivity of the target cell (which is probably higher in BT474 than in SK-BR-3) and on the antibody-triggered immune defense.

Concurrent trastuzumab and IL-15 administration triggered an immune response in BT474- and even in SK-BR-3-based HTM that resulted in tumor eradication and caused reduced metastasis in the lung. These results stress the impact and the importance of immunosurveillance as a vital mechanism for efficient tumor cell elimination, especially during a therapeutic trastuzumab treatment [21]. However, IL-15 induced fatal side effects in both HTM models. More specifically, the cytokine caused a hyper activation of T-cells which most probably contributed to reduced OS of HTM. Advanced dose-finding studies using HTM might help to identify an IL-15 concentration that efficiently triggers the anti-tumor defense accompanied by tolerable and manageable side effects.

A strong capacity of IL-15 to stimulate T- and NK-cells [22-23] both *in-vitro* and *in-vivo*, which is associated with adverse side effects in the clinical setting, has been previously reported [24]. Side effects (including liver damage) were observed even when non-receptor coupled IL-15 was injected, which is supposed to be less effective than the IL-15/IL-15 Rα complex [29-30]. Another study performed with C57BL/6 mice and using murine IL-15/IL-15 Rα complex revealed the immunotoxicity of systemic IL-15 as well [31]. Notably, the mice displayed extravasation of immune cells into the liver which apparently caused an increased liver damage (AST/ALT) and enhanced mortality. Noteworthy is that preclinical safety trials in rhesus macaque [25] failed to identify any side effects linked to IL-15 administration, even at high dosages. This phenomenon impressively demonstrates that safety studies conducted in primates cannot be directly transferred to the clinical situation. In this respect, NSG mice with a human immune system (HTM) might close the gap between pure murine based *in-vivo* studies and the clinical setting.

Trastuzumab opsonization of target cells is thought to trigger FcγRIIIa-(CD16)-expressing NK-cells. Hence, ADCC is assumed to generally contribute to tumor cell eradication. However, three genomic polymorphisms of the Fc-recognizing receptor have been identified which encode receptor variants characterized by homozygous high (V/V), heterozygous high (V/F), and homozygous low (F/F) affinity [32]. On the one hand, there is some evidence for a higher treatment efficiency of e.g., rituximab against Non-Hodgkin-Lymphomas in the presence of the high affinity V/V FcγRIIIa [33]. On the other hand, a more recent study reported no association between FcγRIIIa polymorphism and the clinical outcome [34]. The impact of FcγRIIIa variants in trastuzumab treated patients is still being debated. Only very few analyses have indicated that immune effector cells expressing the high affinity variant (V/V) of the FcγRIIIa allele mediate ADCC of anti-HER2 IgG1 variants better than cells expressing the phenylalanine encoding allele [35]. To the best of our knowledge, there is only one clinical study [36] indicating a correlation between improved outcome and the expression of the high affinity phenotype (V/V). However, a number of other communications taken from the clinical setting did not corroborate this relationship [37-39]. In fact, there is an *ex-vivo* ADCC study indicating a treatment benefit for metastatic patients in the presence of phenylalanine expression [40].

Trastuzumab treatment of HTM results in a prolonged DFS (but not OS) in heterozygous (V/F) high affinity- compared to homozygous low affinity-bearing mice. This finding is substantiated by an increased NK-cell number and elevated CD16 expression in heterozygous animals. Consequently, trastuzumab apparently causes enhanced ADCC only in the “high affinity setting.”

Notably, tumor cell eradication in trastuzumab/IL-15-treated HTM is not only achieved by NK-cell activation but also by a stimulation and increased infiltration of CD33^+^ myeloid cells (e.g., macrophages). A trastuzumab-induced tumor infiltration by macrophages with phagocytotic activity has been recently demonstrated by Shi et al. [41]. The importance of anti-tumor activity in myeloid cells has been explicitly shown by a depletion of macrophages, which resulted in reduced anti-tumor-efficacy in mouse xenograft tumor models.

Noteworthy, IL-15 treatment ended up in the most severe disease progression in the presence of low affinity FcγRIIIa variants, and triggered NK-cells more efficiently than in the high affinity carriers. Nevertheless, tumor elimination after immune modulation seems to be accomplished by a complex and well-orchestrated response that involves a variety of immune cells (e.g., NK-, T-, and myeloid cells) but does not depend on efficient ADCC alone. For example, IL-15 treatment in HTM caused T-cell activation and tumor eradication (reduced tumor volume in BT474; reduced tumor cell count in SK-BR-3) irrespective of the presence of “low” or “high affinity Fc-receptor cells.”

Besides primary tumor growth, a fatal event of tumor progression is tumor cell dissemination into the bm, which is considered to be a metastasis preceding process. On a related note, we addressed the question of whether trastuzumab treatment prevents dissemination or eliminates DTCs from the bm. Notably, antibody treatment alone did not reduce the bm tumor load in either HTM or TM. In contrast, the combined administration of trastuzumab and IL-15, which leads to immune cell activation as described above, tends to diminish the frequency of DTCs in the bm niche. An IL-15-based immune therapy apparently reduces tumor cell dissemination, or enhances the elimination of already disseminated (dormant) DTCs, which are probably barely affected by conventional cytotoxic treatments or by irradiation.

Finally, we investigated to what extent disseminated tumor cells in HTM switch their phenotype and thereby develop trastuzumab insensitivity. In some SK-BR-3-transplanted mice we found DTCs in the bm that showed decreased CD24 and increased CD44 expression compared to the original cells (i.e., solid tumor cells or non transplanted SK-BR-3 cells). In contrast to never transplanted, original SK-BR-3 cells, these CD24^low^/CD44^high^ cells became insensitive to trastuzumab treatment. An enhanced CD44 expression has already been associated with trastuzumab resistance [42-43]. However, its importance as an indicator for tumor stemness is still being debated [44-45]. Interestingly, the development of trastuzumab resistance and CD44 upregulation in DTCs was independent of prior trastuzumab treatment and the presence of an immune system (i.e., immune surveillance). Notably, not all bm cultures with enhanced CD44 expression became trastuzumab resistant, which indicates that increased CD44 expression is somehow associated with, but not solely sufficient for the development of trastuzumab resistance. Thus, elevated CD44 expression in tumor cells in HTM and TM is linked to dissemination / metastases but not necessarily to trastuzumab resistance.

Overall we elucidated the importance of a human immune response for an efficient trastuzumab treatment in HTM, an association of tumor cell phenotype to treatment response, and the power and peril of IL-15 as an immunotherapeutic add-on in cancer. Speculatively, an optimized concentration or a local accumulation of IL-15 by using antibody drug conjugate (ADC) could utilize the beneficial therapeutic capacity without causing serious side effects.

## MATERIAL AND METHODS

### Breast cancer cell lines

BT474 (isolated by Lasfargues and Coutinho; ATCC number HTB-20) or SK-BR-3 (isolated by Trempe and Old; ATCC number HTB-30) breast cancer cells were used for cotransplantation.

### Mice

Humanized tumor mice were generated as previously described [23]. Briefly, NOD-*scid IL2Rγ*^null^ (NSG) mice were obtained from Jackson Laboratories and housed in a specialized pathogen-free facility at the University of Regensburg. Newborn animals were irradiated (1 Gy) during the first 48 hours of life and 3 hours later transplanted with 2.5x10^5^ human CD34^+^ cells isolated from umbilical cord blood (CB) together with 3x10^6^ BT474 or SK-BR-3 tumor cells in the liver.

### Ethic statements

The animal work was approved by the local veterinary authorities of the district government based on the European guidelines and national regulations of the German Animal Protection Act (approval no. 54-2532.1-27/11).

Cord blood samples were taken with approval from the Ethics Committee of the University of Regensburg (permission no. 11-101-0287). All patients included in the study provided written informed consent.

### Trastuzumab +/- IL-15R-alpha complex preparation and treatment

BT474 (age 11 weeks) and SK-BR-3 (age 9 weeks) received 20μg/kg of trastuzumab (Roche Diagnostics, Penzberg, Germany) i.p. every week. IL-15 treatment was performed every second week i.p. (2.5 μg of IL-15/IL-15Ra/Fc complex i.p. in 200 μl of PBS) using human recombinant IL-15 and a recombinant fusion protein consisting of the ectodomain of the human IL-15 receptor-alpha-chain and the human IgG1 Fc (IL-15Ra/Fc; R&D Systems, Minneapolis, USA). The IL-15/IL-15Ra/Fc complex was prepared as previously described [23].

### Mononuclear cell isolation from different tissues

Mononuclear cells were isolated from different mouse tissues as previously described [23]. Briefly, spleen, peritoneal effusion cells (PEC), lung, and liver cells were passed through 40 μm cell strainer (BD Bioscience, USA). Lung and liver cells were resuspended in 5 ml of 40% Percoll/RPMI, underlaid with 5 ml 70% Percoll/RPMI and centrifuged for 20 minutes at 800 x g. Cells were collected from the interface and washed twice. PEC were harvested from the peritoneum after cervical dislocation using 10 ml PBS for perfusion. To collect bone marrow cells, femurs were removed, ends were clipped off and rinsed with 20 ml PBS + 2 mM EDTA using a syringe with a 27 G needle (BD Bioscience, Franklin Lakes, NJ, USA). The resulting MNC suspensions were characterized by flow cytometry or cultured (bone marrow).

### FcgRIIIa-158V-F polymorphism genotyping

Genomic DNA was extracted using QIAamp® DNA Mini and Blood Mini Kit (Qiagen, Hilden, Germany) and the DNA concentrations were measured using a NanoDrop Spectrophotometer ND-1000 (peqLab Biotechnologie, Erlangen, Germany). For genotype analyses, DNA samples were diluted in nuclease-free water to reach a final concentration of 2.5 ng/μl.

Samples were genotyped as published previously by Wu et al. [46] but adapted to enhance FcgRIIIa specificity (e.g. new forward primer (PCR1) was designed). The following primers were used in nested PCR 1 (N1_forward_new: 5’- TAA ATT ACT TGG TGA CAT GAT CG-3’; N1_reverse: 5’- CAG TTG GTA CCC AGG TTG AA-3’) and nested PCR 2 (N2_forward: 5’- ATC AGA TTC GAT CCT ACT TCT GCA GGG GGC AT-3’; N2_reverse: 5’- ACG TGC TGA GCT TGA GTG ATG GTG ATG TTC AC-3’). The first PCR cycles for the nested PCR1 consisted of 5 minutes denaturation, 1½ minutes primer annealing at 56°C, and 2 minutes elongation at 72°C. This was followed by 35 cycles of a reduced denaturation time of 1 minute, and a final cycle of 8 minutes elongation time at 72°C. The cycles for the second nested PCR were identically performed as nested PCR1 but with one minute annealing time at 64°C and one minute of elongation at 72°C.

PCR was performed using GoTaq® Colorless Master Mix (Promega, Mannheim, Germany) and the PCR product was digested with Fast Digest Hin1 II (Thermo Fisher Scientific, Bremen, Germany) for 15 minutes at 37°C and subsequently heat inactivated for 5 minutes at 80°C. Three DNA samples representing FcgRIIIa-158V/V, V/F and F/F genotypes were kindly provided by Dr. Anja Lux (Institute of Genetics, Department of Biology, University of Erlangen-Nuremberg, Germany), and were used to establish the PCR and included in each PCR run as an internal control. The product was analyzed on a 5.0% sieve agarose gel (Biozym, Oldendorf, Germany) including the GelRed Nucleic Acid Stain (Biotium, Cologne, Germany).

### Flow cytometry analysis

The characterization of human immune cells and tumor cells was performed by flow cytometry using a FACSCanto-II flow cytometer which was run by Diva software (Ver. 7.0, BD Biosciences, San Jose, CA, USA). Samples were stained using the following antibodies: anti-CD3-FITC (clone HIT3a), anti-CD19-PE (clone HIB19), anti-CD33-PerCP-Cy5.5 (clone P67.6), anti-CD45-APC (clone HI30), anti-CD56-V450 (clone B159), anti-Nkp46-APC (clone 9E2/Nkp46), anti-CD4 FITC (clone SK3), anti-CD8-PE (clone HIT8a), and anti-HER2-PE (clone Neu 24.7) from BD Biosciences; anti-CD27 PeCy7 (clone O323) from eBioscience (Frankfurt, Germany); anti-CD16 PE (clone 368), anti-CD24 AF647 (clone ML5), anti-CD44 AF488 (clone IM7), and anti-EpCAM BV421 (clone 9C4) from BD BioLegend (San Diego, CA, USA). Appropriate mouse immunoglobulin antibodies were used as isotype controls for all stainings.

### Immunohistochemistry and imaging

Tissue samples of spleen, liver, lung, brain and tumor were fixed in 4% formalin, embedded in paraffin and stained as previously described [29].

The following antibodies from the ultraView Universal DAB Detection Kit (Ventana, Roche) were used for (I) the humanized mice: anti-CD4 (SP35), anti-CD8 (SP57), anti-CD79a (SCB117), anti-CD68 (PG-MI), as well as anti CD44 (clone DF1485; Dako) and polyclonal rabbit anti HER2 (order number A0485, Dako). All antibodies were tested for specificity using tumor mice without human immune cells and humanized mice without co-transplanted tumor cells. In general the same positive controls as used in routine diagnostics were also used for this study. Histological specimens were imaged with an AxioImager Z1 microscope (Zeiss, Oberkochen, Germany).

### Bone marrow cell culture and flow cytometric cell cycle analysis

For enrichment of the low number of cancer cells in organs of HTM and TM, single cell suspensions from bone marrow were cultured in RPMI Medium 1640, and supplemented with 10% fetal calf serum and antibiotics (100 units/ml penicillin, 100 μg/ml streptomycin). Cells were harvested after cell expansion (2-14 weeks) by trypsination and analyzed by flow cytometry. For trastuzumab resistance studies, cells were plated for ninety-six hours in 6-well plates, treated for 48 hours with 5 μg/ml trastuzumab (Roche Diagnostics, Penzberg, Germany) and the proliferation capacity was analyzed as follows: Harvested cells were washed twice with ice-cold PBS containing 2% FCS, then fixed and permeabilized with 70% methanol on ice over night. Finally, cells were washed twice with PBS and incubated in the presence of 10 μg/ml RNAse for 30 minutes at 37°C. The DNA intercalating 4′,6-Diamidin-2-phenylindol (DAPI) fluorochrome was added at a final concentration of 1 μg/ml 15 min prior to analysis to ensure quantitative DNA staining. The DNA dye DAPI was excited with the violet excitation line and fluorescence emission was detected by the optical trigon unit equipped with a 450/50 bp filter. 3x10^5^ DAPI stained cells of every sample were collected. DNA histograms were plotted on a linear scale upon cell doublet, aggregate, and debris discrimination via pulse processing. Cell cycle fractions, i.e., percentages of cells in G0/G1-, S- and G2/M-phase, were quantified using ModFit LT 3.2 software (Verity Software House, Topsham, ME, USA). Treatment effects are expressed by the reduction in the S-phase fraction compared to untreated cells.

## SUPPLEMENTARY MATERIALS FIGURES


