# Supplementary material for: IL-15 enhances the anti-tumor activity of trastuzumab against breast cancer cells but causes fatal side effects in humanized tumor mice (HTM)
Source: Oncotarget. 2016 Jul 11;8(2):2731–44. doi: 10.18632/oncotarget.13159 (PMC5356837; doi:10.18632/oncotarget.13159)
Supplement: Supplementary file 1 [file oncotarget-08-2731-s001.pdf]

IL-15 enhances the anti-tumor activity of trastuzumab against breast cancer cells but causes fatal side effects in humanized tumor mice (HTM)

SUPPLEMENTARY FIGURES

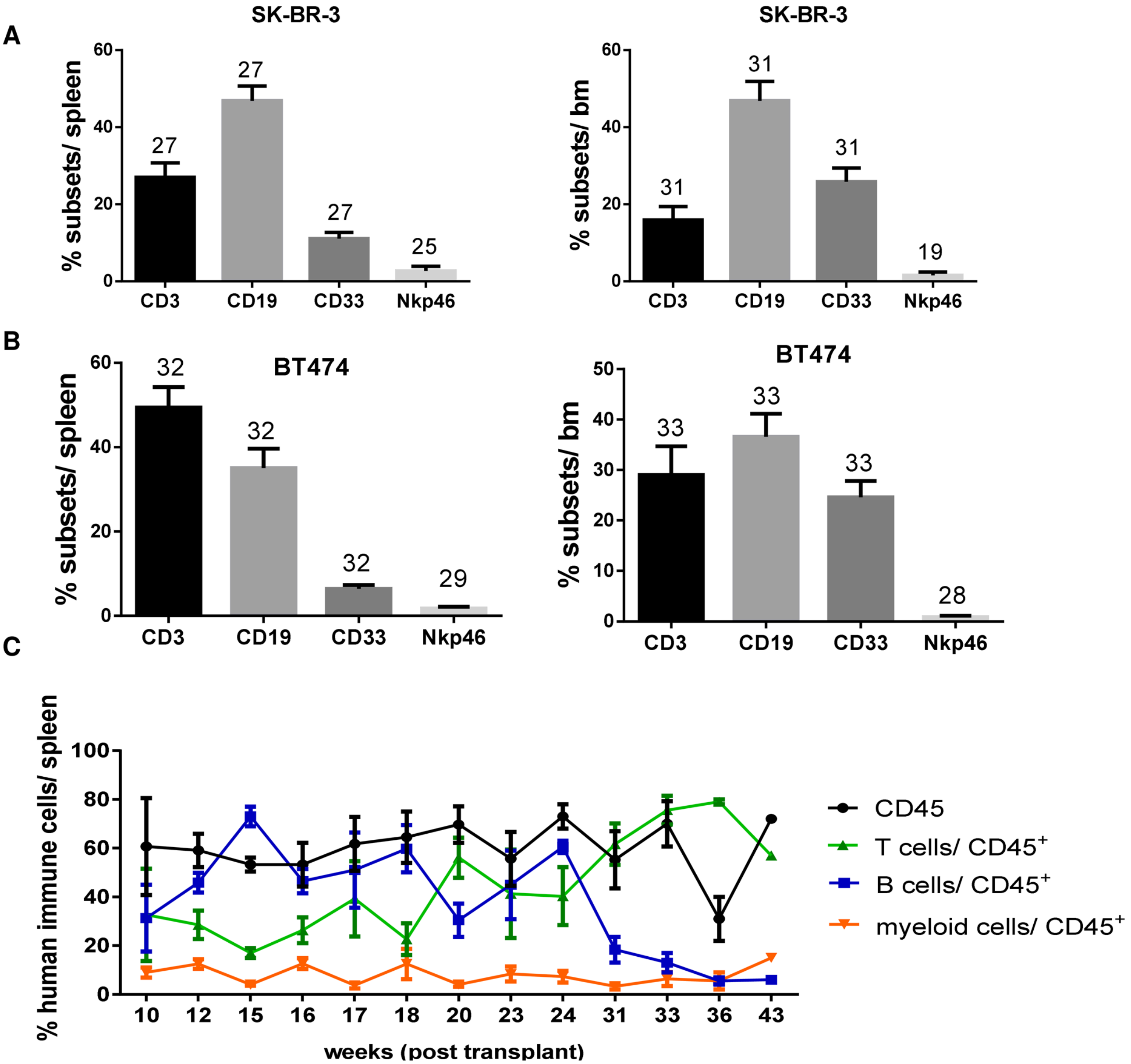

Supplementary Figure S1: Human immune cell reconstitution in spleen and bm of HTM. Flow cytometric analyses of T- (CD3), B- (CD19), and myeloid (CD33) cells in the spleen and the bone marrow (bm) of **(A)** SK-BR-3 (age: 16 weeks +/- 0.8 (mean +/- SEM)) and **(B)** BT474 (age: 23 weeks +/- 1.3 (mean +/- SEM)) transplanted HTM. The number of animals included in each analysis is mentioned above each bar. **(C)** Percentage of total human immune cells (CD45), T (CD3), B (CD19), and myeloid (CD33) cells in the spleen of HTM over time.

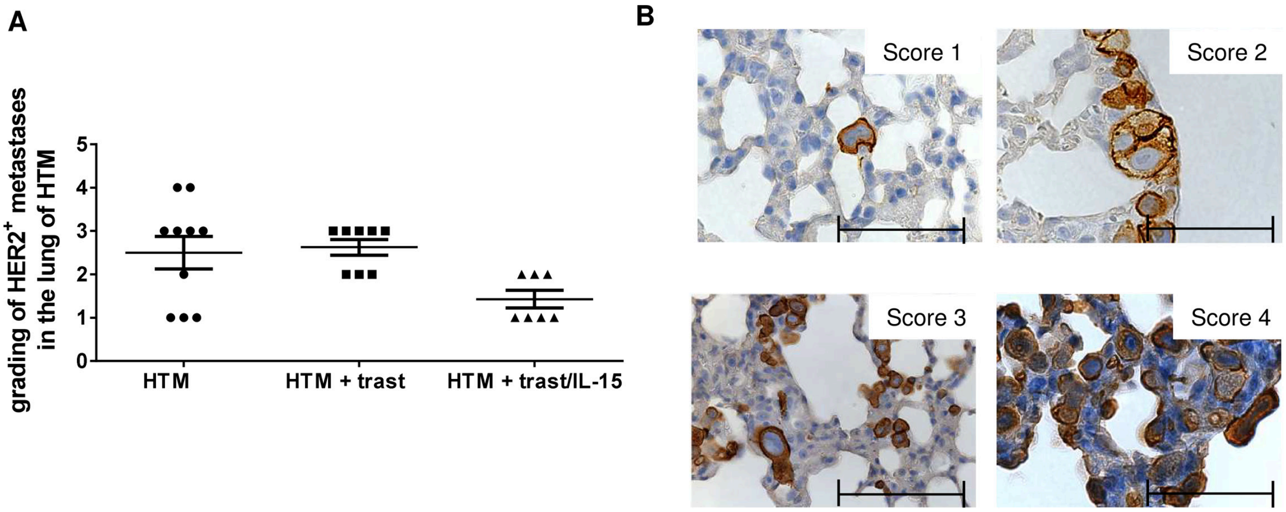

Supplementary Figure S2: The effect of IL-15 substitution on lung metastases in SK-BR-3 HTM. **(A)** Illustration of the individual results of lung metastases grading in treated (trastuzumab +/- IL-15) and non-treated (control) SK-BR-3-transplanted HTM. **(B)** Exemplary illustration of the classification of the lung metastases score in SK-BR-3-transplanted animals (score 1= single tumor cells; score 2 = single metastasis; score 3 = small metastases covering the whole lung; score 4 = larger tumor cell clusters covering the whole lung). Bars represent 100 µm.

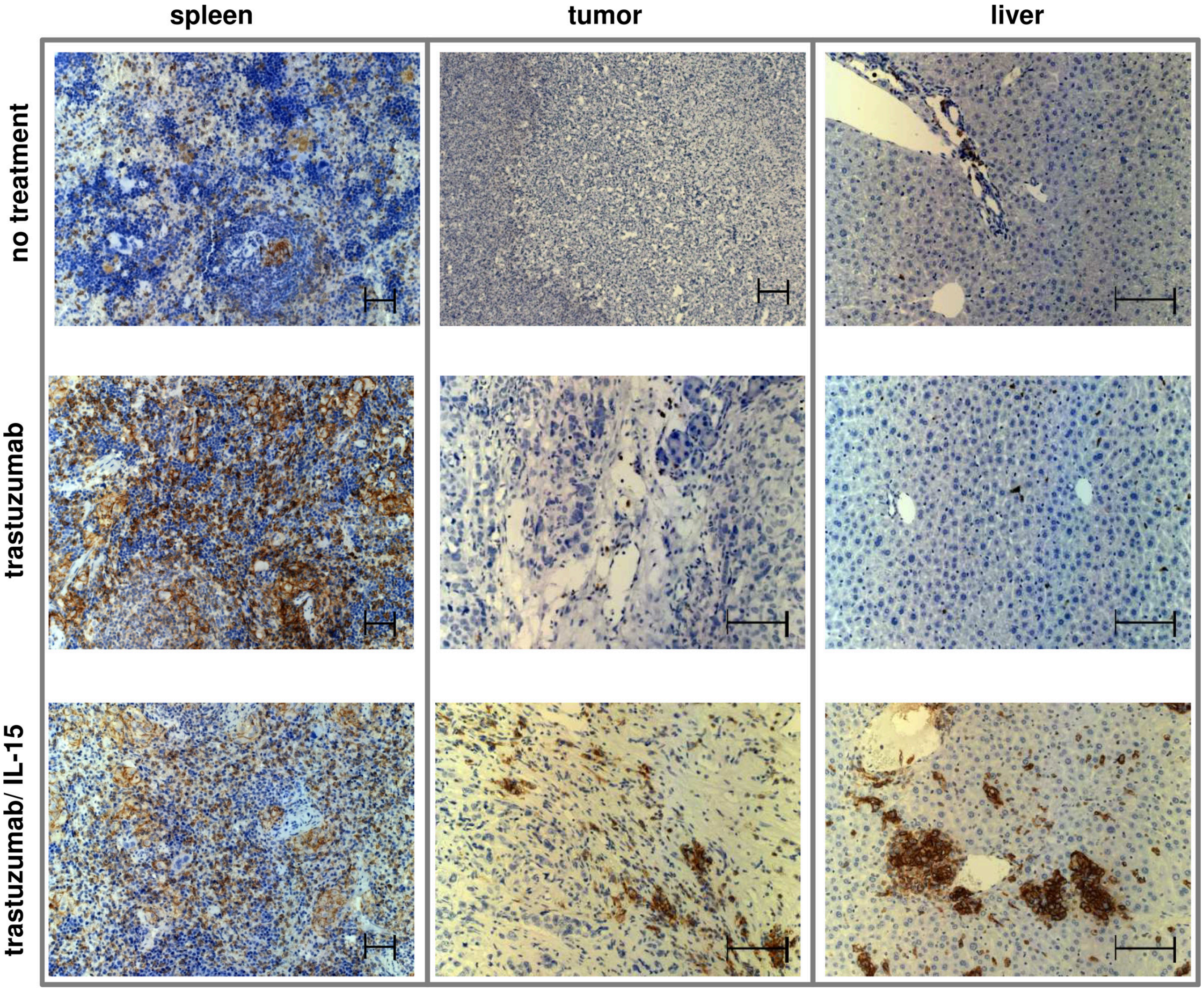

Supplementary Figure S3: CD44 expression profile in different organs in untreated and treated (IL-15 +/-trastuzumab) BT474-transplanted HTM. Spleen, tumor and liver of HTM with or without treatment were immunhistologically stained for CD44 expression. Bars indicate 100 µm.

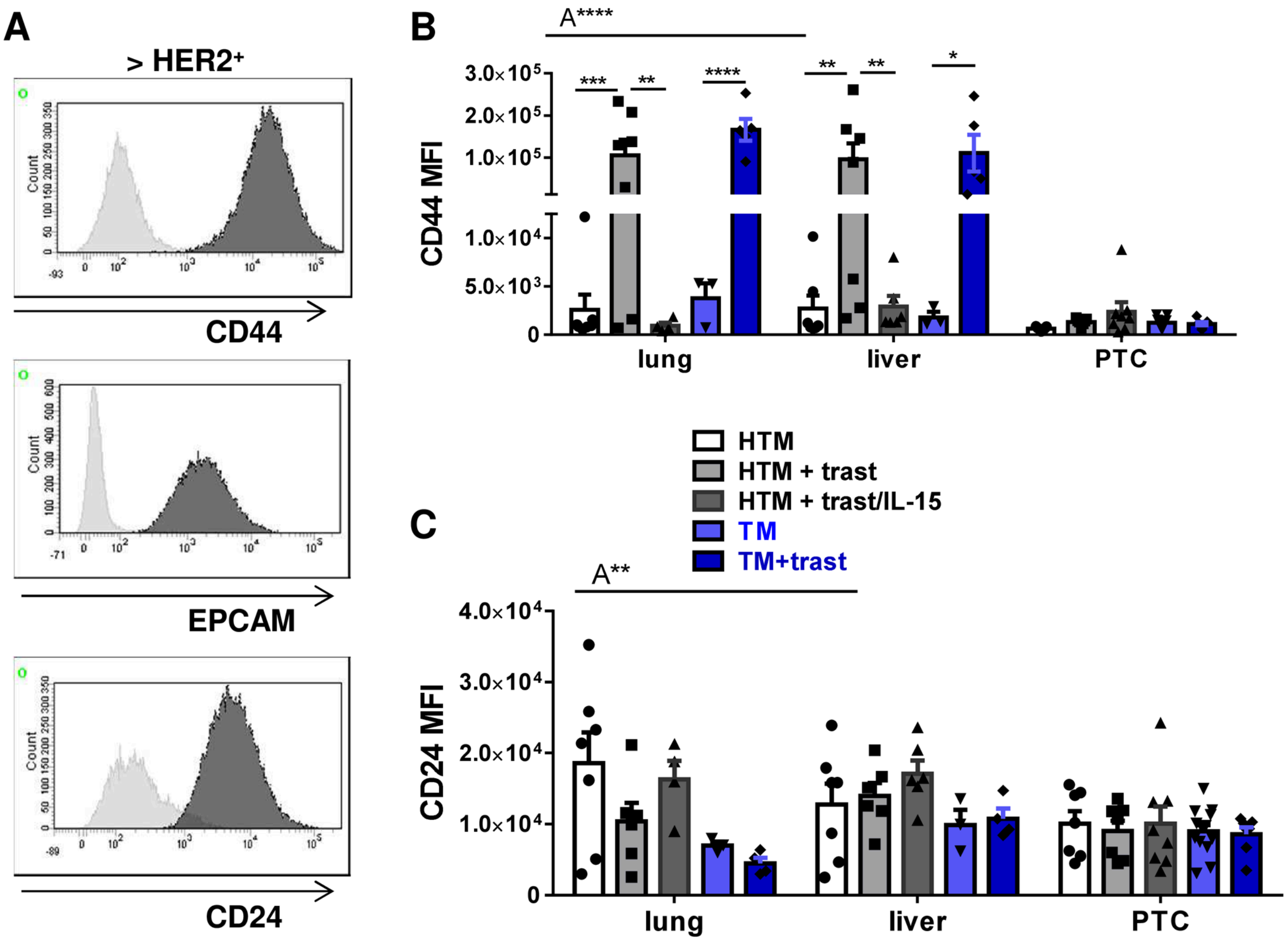

Supplementary Figure S4: Flow cytometric analyses of the tumor cell phenotype in lung, liver, and PTC of treated and untreated SK-BR-3-transplanted HTM and TM. Single cells from lung, liver, and peritoneal cavity (PTC) of the animals were isolated and stained for HER2, CD44, EpCAM, and CD24 expression **(A)**. The mean fluorescence (MFI) of CD44 **(B)** and CD24 **(C)** of HER2+ cells (>HER2$^+$) were analyzed and calculated using two-way Anova (A**=p<0.01; A****=p<0.0001) and Tukey's multiple comparison test (*=p<0.05; **=p<0.01; ***=p<0.001; ****=p<0.0001). Bars represent the mean +/- SEM. Each symbol represents one single animal.
